# Efficacy of Alpha Lipoic Acid Supplementation in Sperm Parameters: A Systematic Review and Meta-Analysis of Randomized Trials

**DOI:** 10.1590/S1677-5538.IBJU.2024.0614

**Published:** 2025-04-20

**Authors:** Iago Zang Pires, Marília Oberto da Silva Gobbo, Renan Yuji Ura Sudo, Tanize Louize Milbradt, Nilson Marquardt, Gustavo Franco Carvalhal, Carlos Teodosio Da Ros

**Affiliations:** 1 Universidade Católica do Rio Grande do Sul Departamento de Medicina PUCRS RS Brasil Departamento de Medicina, Pontifícia Universidade Católica do Rio Grande do Sul – PUCRS, RS, Brasil;; 2 Universidade Federal da Grande Dourados Departamento de Medicina Dourados MS Brasil Departamento de Medicina, Universidade Federal da Grande Dourados - UFGD, Dourados, MS, Brasil;; 3 Universidade Federal de Santa Maria Departamento de Medicina Santa Maria RS Brasil Departamento de Medicina, Universidade Federal de Santa Maria - UFSM, Santa Maria, RS, Brasil;; 4 Universidade Católica do Rio Grande do Sul Hospital São Lucas Departamento de Urologia PUCRS RS Brasil Departamento de Urologia, Hospital São Lucas, Pontifícia Universidade Católica do Rio Grande do Sul – PUCRS, RS, Brasil;; 5 Universidade Luterana do Brasil Disciplina de Urologia Canoas RS Brasil Disciplina de Urologia, Universidade Luterana do Brasil – ULBRA, Canoas, RS, Brasil

**Keywords:** Infertility, Male, Antioxidants, alpha-lipoic acid

## Abstract

**Introduction:**

Male factors contribute to 30 to 50% of infertility in couples. Treatment options for male infertility are limited, so antioxidant supplementation for idiopathic male infertility is currently being studied. Alpha lipoic acid (ALA) has a high antioxidant capacity and the potential to penetrate tissues, cells, and organelles, including mitochondria, due to its water and lipid solubility properties. The recent inclusion of randomized trials in the literature has required a new systematic review and meta-analysis to evaluate the efficacy of alpha lipoic acid in sperm parameter changes.

**Purpose:**

We aimed to perform a systematic review and meta-analysis of the currently available randomized trials comparing the effects of ALA supplementation versus placebo on sperm function in infertile male patients.

**Material and Methods:**

Pubmed, Embase, Cochrane Library, and Scopus databases were searched from inception to June 2024. A random-effects model was employed to compute mean differences and risk ratios for continuous and binary endpoints. Heterogeneity was evaluated through the prediction interval. A sensitivity analysis was conducted by systematically excluding one study at a time and recalculating the pooled effect. All statistical analysis was conducted using R software 4.4.1. The certainty of evidence was evaluated with the GRADE approach. Results were reported following the PRISMA statement guidelines. This study was registered in PROSPERO.

**Results:**

Five randomized trials comprising 250 patients with a mean age of 28 to 40 years were included in this analysis. Over a mean follow-up time of 3 months, ALA was associated with a reduced proportion of abnormal sperm morphology (MD −0.89; 95% CI −1.48 to −0.29; p=0.003), increased total motility (MD 13.49; 95% CI 3.52 to 23.46; p=0.008), and increased sperm progressive motility (MD 12.43; 95% CI 2.89 to 21.97; p=0.01). Additionally, ALA was associated with a higher pregnancy rate in two individual studies reporting the outcome, however, no significance was found in our pooled analysis (RR 2.28; 95% CI 0.66 to 7.85; p=0.1). Finally, ALA did not change ejaculation volume (MD 0.14; 95% CI −0.54 to 0.83; p=0.6), sperm concentration (MD 11.99; 95% CI −0.67 to 24.66; p=0.06), live sperm (MD 4.42; 95% CI −3.17 to 12.02; p=0.2), or total antioxidant capacity (MD 0.43; 95% CI −0.02 to 0.87; p=0.06). No adverse events were reported.

**Conclusion:**

In this meta-analysis, ALA was associated with a favorable change in sperm quality. However, there were no effects on pregnancy rates. ALA should be considered for patients with idiopathic infertility.

## INTRODUCTION

Almost 15% of couples are unable to conceive after one year of unprotected intercourse and seek medical treatment for infertility. A male factor is solely responsible for about 30-50% of involuntary childless couples and usually are associated with abnormal semen parameters (1, 2). Male infertility occurs due to a variety of conditions. Some of these are identifiable and reversible, such as varicocele and infection. Certain conditions can be identified and treated, but they remain irreversible, such as genetic disorders. The etiology of an abnormal semen analysis (SA) is not identified in approximately 30% of men in which case this condition is termed idiopathic male infertility. This circumstance may be associated with several previously unidentified pathological factors, which promote endocrine disruption due to environmental pollution, generation of reactive oxygen species, sperm DNA damage, or genetic and epigenetic abnormalities. On several occasions, patients with normal SA have sperm that do not function in a manner necessary for fertility (3).

There are limited treatment options available for male infertility. However, it is believed that vitamin supplementation, nutritional formulations, and antioxidants could improve conception rates. Moreover, oxidative stress is considered a major factor in the pathogenesis of idiopathic infertility. It decreases sperm motility and increases reactive oxygen species (ROS), which impair sperm function (4, 5). Therefore, seminal levels of ROS have been negatively associated with conception outcomes. One of the most studied antioxidants is alpha lipoic acid (ALA), which has water- and fat-soluble properties that propitiate high antioxidant capacity and the potential to penetrate tissues, cells, and organelles, including mitochondria (6).

ALA has singular antioxidant qualities, working effectively in both its oxidized and reduced forms. This antioxidant, with its low molecular mass and dual solubility, can regenerate endogenous antioxidants, such as vitamins E and C, and neutralize free radicals. It also has scavenging activity and metal-chelating properties (7). In this way, ALA can help preserve the vitality and motility of sperm by reducing the production of ROS. In addition, ALA has been proven to improve sperm quality and maintain sperm function by reducing lipid peroxidation and increasing the activity of antioxidant enzymes (8).

Given the promising results observed in studies on ALA, and the recent inclusion of randomized trials, a new systematic review and meta-analysis is essential to evaluate the efficacy of alpha lipoic acid in sperm parameter changes and pregnancy rates.

## MATERIAL AND METHODS

### Search strategy and data extraction

This systematic review and meta-analysis adopts the recommendations of the Cochrane Collaboration and it is presented following the Preferred Reporting Items for Systematic Reviews and Meta-Analysis (PRISMA) guideline (Table-S1) (9, 10). Before the literature screening, the study protocol was prospectively registered on June 28, 2024, in the PROSPERO database with the identification number CRD42024564932. Two authors (I.P. and R.S.) performed independent and systematic searches in PubMed, Embase, Scopus and the Cochrane Central Register of Controlled Trials from inception to July 2024, using the following search terms: ‘spermatozoa’, ‘semen’, ‘sperm’, ‘male infertility’, ‘male subfertility’, ‘asthenozoospermia’, ‘asthenospermia’, ‘oligozoospermia’, ‘oligospermia’, ‘oligoasthenozoospermia’, ‘oligoasthenospermia’, ‘oligoasthenoteratozoospermia’, ‘oligoasthenoteratospermia’, ‘OAT’, ‘teratozoospermia’, ‘teratospermia’, ‘spermatogenesis’, ‘oral alpha-lipoic acid’, ‘alpha-lipoid acid’, ‘alpha lipoic acid’, ‘α-lipoid acid’, ‘thioctic acid’, ‘randomized trials’, ‘randomized control trial’, "RCT". The search strategy was filtered for the years 2000 to 2024. No language filter was used. The entire and detailed search strategy is provided in the Supplementary Material. Additionally, the references of all included studies were manually searched to identify any further relevant studies. Three authors (I.Z. M.G., R.S.) independently extracted data following predefined search criteria and quality assessment. Discordances were solved through discussion with two additional authors (T. M. and N. M.) and, when necessary, through consultation with the senior author.

**Table S1:** PRISMA checklist.

Section and Topic	Item #	Checklist item	Location where item is reported
TITLE			
Title	1	Identify the report as a systematic review.	
ABSTRACT			
Abstract	2	See the PRISMA 2020 for Abstracts checklist.	
INTRODUCTION			
Rationale	3	Describe the rationale for the review in the context of existing knowledge.	
Objectives	4	Provide an explicit statement of the objective(s) or question(s) the review addresses.	
METHODS			
Eligibility criteria	5	Specify the inclusion and exclusion criteria for the review and how studies were grouped for the syntheses.	
Information sources	6	Specify all databases, registers, websites, organisations, reference lists and other sources searched or consulted to identify studies. Specify the date when each source was last searched or consulted.	
Search strategy	7	Present the full search strategies for all databases, registers and websites, including any filters and limits used.	
Selection process	8	Specify the methods used to decide whether a study met the inclusion criteria of the review, including how many reviewers screened each record and each report retrieved, whether they worked independently, and if applicable, details of automation tools used in the process.	
Data collection process	9	Specify the methods used to collect data from reports, including how many reviewers collected data from each report, whether they worked independently, any processes for obtaining or confirming data from study investigators, and if applicable, details of automation tools used in the process.	
Data items	10a	List and define all outcomes for which data were sought. Specify whether all results that were compatible with each outcome domain in each study were sought (e.g. for all measures, time points, analyses), and if not, the methods used to decide which results to collect.	
	10b	List and define all other variables for which data were sought (e.g. participant and intervention characteristics, funding sources). Describe any assumptions made about any missing or unclear information.	
Study risk of bias assessment	11	Specify the methods used to assess risk of bias in the included studies, including details of the tool(s) used, how many reviewers assessed each study and whether they worked independently, and if applicable, details of automation tools used in the process.	
Effect measures	12	Specify for each outcome the effect measure(s) (e.g. risk ratio, mean difference) used in the synthesis or presentation of results.	
Synthesis methods	13a	Describe the processes used to decide which studies were eligible for each synthesis (e.g. tabulating the study intervention characteristics and comparing against the planned groups for each synthesis (item #5)).	
	13b	Describe any methods required to prepare the data for presentation or synthesis, such as handling of missing summary statistics, or data conversions.	
	13c	Describe any methods used to tabulate or visually display results of individual studies and syntheses.	
	13d	Describe any methods used to synthesize results and provide a rationale for the choice(s). If meta-analysis was performed, describe the model(s), method(s) to identify the presence and extent of statistical heterogeneity, and software package(s) used.	
	13e	Describe any methods used to explore possible causes of heterogeneity among study results (e.g. subgroup analysis, meta-regression).	
	13f	Describe any sensitivity analyses conducted to assess robustness of the synthesized results.	
Reporting bias assessment	14	Describe any methods used to assess risk of bias due to missing results in a synthesis (arising from reporting biases).	
Certainty assessment	15	Describe any methods used to assess certainty (or confidence) in the body of evidence for an outcome.	
RESULTS			
Study selection	16a	Describe the results of the search and selection process, from the number of records identified in the search to the number of studies included in the review, ideally using a flow diagram.	
	16b	Cite studies that might appear to meet the inclusion criteria, but which were excluded, and explain why they were excluded.	
Study characteristics	17	Cite each included study and present its characteristics.	
Risk of bias in studies	18	Present assessments of risk of bias for each included study.	
Results of individual studies	19	For all outcomes, present, for each study: (a) summary statistics for each group (where appropriate) and (b) an effect estimate and its precision (e.g. confidence/credible interval), ideally using structured tables or plots.	
Results of syntheses	20a	For each synthesis, briefly summarise the characteristics and risk of bias among contributing studies.	
	20b	Present results of all statistical syntheses conducted. If meta-analysis was done, present for each the summary estimate and its precision (e.g. confidence/credible interval) and measures of statistical heterogeneity. If comparing groups, describe the direction of the effect.	
	20c	Present results of all investigations of possible causes of heterogeneity among study results.	
	20d	Present results of all sensitivity analyses conducted to assess the robustness of the synthesized results.	
Reporting biases	21	Present assessments of risk of bias due to missing results (arising from reporting biases) for each synthesis assessed.	
Certainty of evidence	22	Present assessments of certainty (or confidence) in the body of evidence for each outcome assessed.	
DISCUSSION			
Discussion	23a	Provide a general interpretation of the results in the context of other evidence.	
	23b	Discuss any limitations of the evidence included in the review.	
	23c	Discuss any limitations of the review processes used.	
	23d	Discuss implications of the results for practice, policy, and future research.	
OTHER INFORMATION			
Registration and protocol	24a	Provide registration information for the review, including register name and registration number, or state that the review was not registered.	
	24b	Indicate where the review protocol can be accessed, or state that a protocol was not prepared.	
	24c	Describe and explain any amendments to information provided at registration or in the protocol.	
Support	25	Describe sources of financial or non-financial support for the review, and the role of the funders or sponsors in the review.	
Competing interests	26	Declare any competing interests of review authors.	
Availability of data, code and other materials	27	Report which of the following are publicly available and where they can be found: template data collection forms; data extracted from included studies; data used for all analyses; analytic code; any other materials used in the review.	

*From:* Page MJ, McKenzie JE, Bossuyt PM, Boutron I, Hoffmann TC, Mulrow CD, et al. The PRISMA 2020 statement: an updated guideline for reporting systematic reviews. BMJ 2021;372:n71. doi: 10.1136/bmj.n71. This work is licensed under CC BY 4.0. To view a copy of this license, visit https://creativecommons.org/licenses/by/4.0/

### Eligibility criteria

This meta-analysis included studies that strictly complied with the following eligibility criteria: [1] randomized studies; [2] comparing alpha lipoic acid with placebo; [3] involving men of reproductive age, ranging from 23 to 50 years old, with infertility and baseline semen samples that met World Health Organization (WHO) criteria; and [4] with a follow-up period of 2 months or more after the treatment since this treatment period was stipulated by previous animal studies (11). Only articles with 600 mg daily of ALA were selected. In addition, studies should report any relevant clinical outcomes of interest, such as sperm parameters variation, pregnancy, and antioxidant activity. Exclusion criteria included studies with [1] no control group, [2] animal studies, [3] literature reviews, and [4] unavailable full texts. Studies involving combined therapies were excluded to verify only the effects of ALA on sperm parameters and reduce bias.

### Endpoints

Mean values for sperm parameters following treatment with ALA and sham therapy were extracted from each study. These parameters included semen volume, sperm concentration, normal sperm morphology, abnormal sperm forms, total motility (Grade A+B+C), progressive motility (Grade A+B), and vitality. In addition, pregnancy rate, measured by the number of pregnancies that occurred in the study period, and total antioxidant capacity, measured by the number of total antioxidants in seminal plasma, were recorded.

### Quality assessment

The evaluation of RCTs employed the Cochrane Collaboration's risk of bias 2 tool (RoB-2), categorizing studies into high, low, or unclear risk across five domains: selection, performance, detection, attrition, and reporting biases (12). Two independent authors (I.P. and M.G.) adhered to the Grading of Recommendations, Assessment, Development, and Evaluation (GRADE) handbook guidelines to assess the evidence's certainty level, utilizing categorizations ranging from high to very low (13). Disagreements among reviewers during the quality assessment were resolved through discussion with a third author (N.M.).

## Statistical analysis

The data were summarized using a random effects model with a restricted maximum likelihood estimator. Binary outcomes were calculated using the risk ratio (RR), while continuous outcomes were summarized using the mean difference (MD). Statistical significance was established by a 95% confidence interval (CI) and a p-value below 0.05. Heterogeneity analysis was conducted by calculating the prediction interval (PI) associated with the I-squared statistic, Cochrane's Q-test, p-value, and Tau. To better evaluate the influence of individual studies on the overall outcomes, a sensitivity analysis was conducted by systematically excluding one study at a time (leave-one-out method) and recalculating the pooled effect. This approach allowed us to evaluate the robustness of the results and identify potential outliers. All statistical analyses were performed using the R software version 4.3.1 (R Foundation for Statistical Computing) (14).

## RESULTS

### Study selection and characteristics

**Figure 1:**
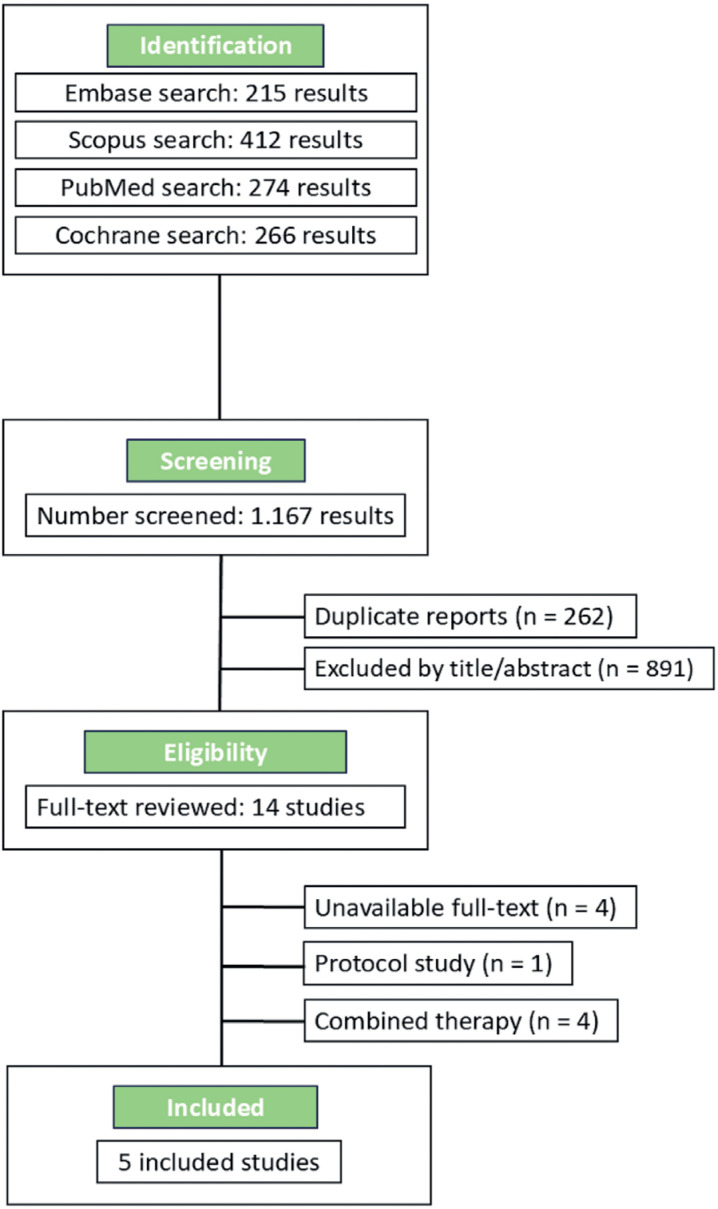
PRISMA flow diagram of study screening and selection.

As illustrated in Figure-1, the initial search provided 1,167 results. After excluding 262 duplicates, 891 articles were excluded based on title and abstract review. Thereafter, 14 studies were fully evaluated. In this comprehensive analysis, four articles were excluded due to the unavailability of the full text, one due to protocol or project analysis and technical specification, and four due to combination therapy use. Finally, five studies published between 2012 and 2023, involving 250 patients with abnormal sperm parameters, were included. We selected randomized trials that compared ALA to a placebo, ensuring that the same dosage of ALA was used across all studies.

Three studies were triple-blind (15–17), one double-blind (18), and one single-blind (19). All the studies used 600 mg daily of ALA. In Raaia et al., the dose of ALA was divided into two doses of 300mg. The course of treatment differed in some studies. In two trials the follow-up was 80 days (16, 17), while two others carried out a 3-month follow-up. Only one study implemented a 12-week follow-up. While there was a difference during treatment, it was minimal - approximately equivalent to one spermatogenic cycle - and did not impact on our statistical results. As part of the treatment, one study performed varicocelectomy (16) on all patients and then compared ALA supplementation with placebo, since one of the inclusion criteria was varicocele. All the other trials used only ALA as an intervention. The semen parameters analyzed in the five studies included sperm concentration, semen volume, total number of sperm, normal forms, abnormal forms of sperm, rapid progressive motility (grade A), slow progressive motility (grade B), non-progressive motility (grade C), immobile spermatozoa (grade D), total motility (grade A+B+C), progressive motility (grade A+B), vitality and pregnancy rate. The baseline characteristics of the included studies are reported in Table-1.

**Table 1:** Key characteristics of the included studies and baseline patient demographics.

Study author/year		Abbasi 2020	Habibi 2023	Haghighian 2015	Hodeeb 2023	Raaia 2012
Design		Triple-blind Randomized Placebo-controlled	Triple-blind Randomized Placebo-controlled	Triple-blind Randomized Placebo-controlled	Single-blind Randomized Case-controlled	Double-blind Placebo-controlled
Follow-up/ Treatment course		80 days	80 days	12 weeks	90 days	3 months
**Concomitant therapy**		Varicocelectomy	None	None	None	None
**Intervention**	**Treatment (n)**	19	25	23	40	24
	**PBO (n)**	22	12	21	40	24
	**Dosage**	Oral ALA (600 mg/day)	Oral ALA (600 mg/day)	Oral ALA (600 mg/day)	Oral ALA (600 mg/day)	Oral ALA (300 mg twice/day)
**Age (y)**	**Treatment (n)**	31	40	33	34	33
	**PBO (n)**	32	37	34	35	28
**Smoking**	**Treatment (n)**	NA	NA	35	NA	54
	**PBO (n)**			33		62
**Weight (kg)** ^§^	**Treatment (n)**	81.25 (74.63–87.86)	79.35(2.69)	88.14 (9.51)	NA	NA
	**PBO (n)**	74.85 (71.65–78.04)	(88.3 (7.45)	89.51 (11.08)		
**Height (cm)**	**Treatment (n)**	179.21 (176.22–182.20)	172.13 (1.17)	177.23 (7.23)	NA	NA
	**PBO (n)**	176.48 (174.60–178.35)^§^	174.7 (1.33)	176.35 (7.15)		
**BMI (kg/m²)**	**Treatment (n)**	25.39 (23.11-27.67)	27.02 (0.82)	28.04 (2.88)	NA	NA
	**PBO (n)**	24.02 (23.08-24.96)^§^	28.76 (2.21)	28.78 (3.39)		
**Ejaculation volume (mL)**	**Treatment (n)**	1.99 (0.28)	4.51 (0.35)	3.59 (0.28)	1.80 (0.64)	NA
	**PBO (n)**	2.31 (0.31)	3.35 (0.3)	3.58 (0.31)	1.76 (0.70)	
**Sperm concentration (10/mL)**	**Treatment (n)**	52.37 (12.55)	60.96 (9.71)	22.63 (1.98)	15.17 (9.6)	30.63 (52.69)
	**PBO (n)**	47.91 (12.16)	78.54 (24.08)	22.83 (2.69)	16.16 (10.18)	23.93 (22)
**Mobility grade a+b+c (%)**	**Treatment (n)**	36.41 (5.61)	49.62 (4.2)	35.1 (3.78)	11.41 (5.45)	27.96 (9.8)
	**PBO (n)**	38.38 (5.71)	51.91 (7.05)	35.32 (4.03)	10.15 (6.87)	28.62 (12.91)
**Abnormal morphology (%)**	**Treatment (n)**	97.21 (0.39)	98.08 (0.31)	15.58 (3.6)	3.97 (1.33)	71.29 (14.82)
	**PBO (n)**	98.27 (0.33)	97.09 (0.87)	14.64 (3.1)	3.73 (1.34)	69.2 (16.1)
**Live sperm (%)**	**Treatment (n)**	NA	78.91 (3.67)	71.85 (3.72)	61.79 (13.45)	NA
	**PBO (n)**		83.20 (6.4)	73.52 (4.2)	58.62 (14.91)	
**Sperm progressive motility (%)**	**Treatment (n)**	23.70 (3.44)	NA	27.97 (2.99)	11.65 (3.54)	18.73 (8.44)
	**PBO (n)**	24.86 (4.09)		27.55 (2.40)	13.24 (4.36)	20.9 (10.6)

Values are presented in mean (SD) or absolute numbers (N) unless otherwise specified. ALA = Alpha lipoic acid; BMI = Body Mass Index; PBO = Placebo; NA: not available; Median (IQR)

### Pooled analysis of all studies

**Figure 2:**
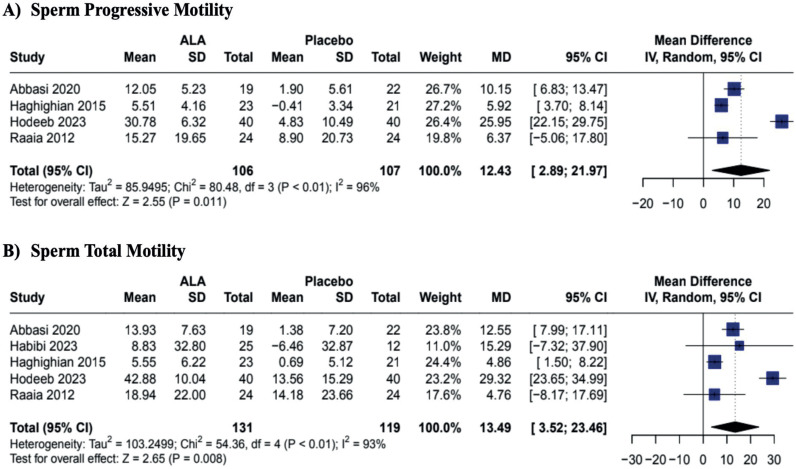
Effects of ALA on Sperm Motility.

**Figure 3:**
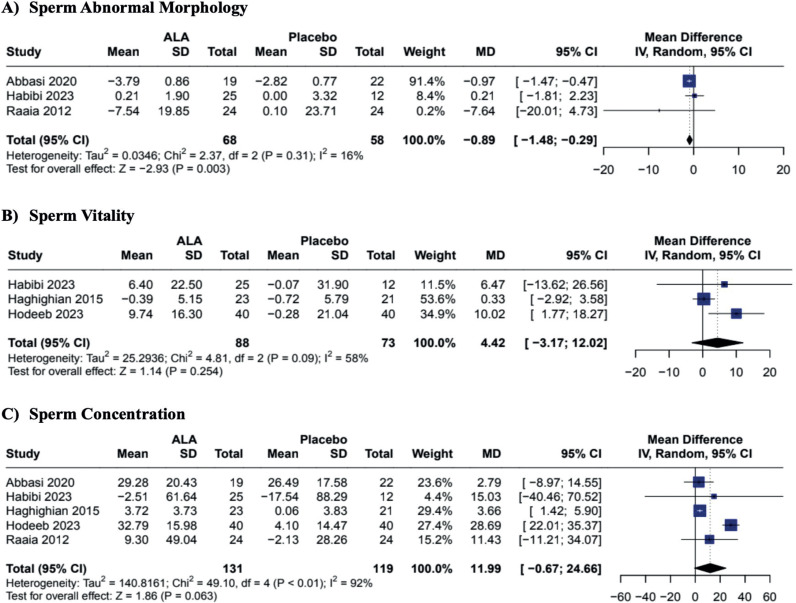
Effects of ALA on Sperm Morphology, Vitality and Concentration.

**Figure 4:**
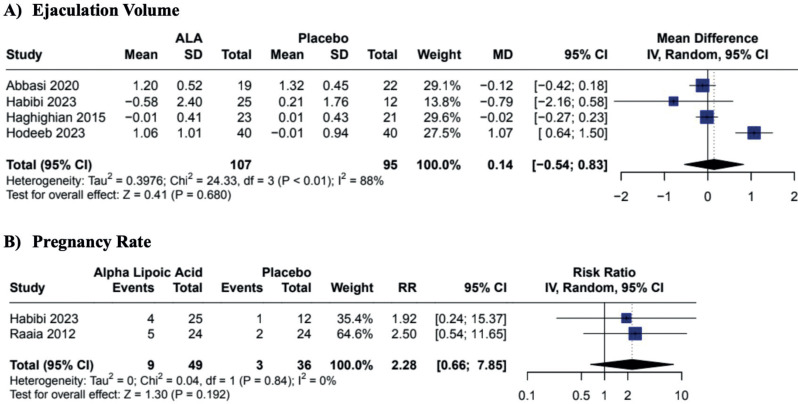
Effects of ALA on Ejaculation Volume and Pregnancy Rates.

In our pooled analysis, the oral supplementation of ALA when compared to placebo demonstrated improvement in sperm progressive motility (MD 12.43; CI 95% 2.89 to 21.97; p = 0.011; I2 = 96%; Figure-2A) and sperm total motility (MD 13.49; CI 95% 3.52 to 23.46; p = 0.008; I2 = 93%; Figure-2B). Additionally, ALA was associated with a reduction of sperm abnormal morphology (MD −0.89; CI 95% −1.48 to −0.29; p = 0.003; I2 = 16%; Figure-3A). The antioxidant supplementation did not change the sperm vitality or ejaculation volume when compared to placebo (MD 4.42; CI 95% −3.17 to 12.02; p = 0.254; I2 58%; Figure-3B; and MD 0.14; CI 95% −0.54 to 0.83; p = 0.680; I2 = 88%; Figure-4A, respectively). There was a slight improvement in sperm concentration, but it was non-statistically significant (MD 11.99; CI 95% −0.67 to 24.66; p = 0.063; I2 = 92%; Figure-3C). Only two studies analyzed the pregnancy rates, demonstrating that the supplementation was not associated with pregnancy rate changes (MD 2.28; CI 95% 0.66 to 7.85; p = 0.192; I2 = 0%; Figure-4B).

### Leave-one-out analysis

Three outcomes, "Sperm progressive motility", "Sperm total motility" and "Sperm concentration" showed high heterogeneity. To investigate this variability, a leave-one-out sensitivity analysis was performed (Figure-S1 A-F). After sequentially excluding one study at a time, it was observed that the treatment benefit remained consistent. Regarding the outcome "Sperm concentration", the I² showed a significant value of 91.8%. Notably, the study conducted by Hodeeb et al. contributed significantly to the observed heterogeneity. After excluding this study, the heterogeneity index (I²) decreased to 0%, indicating a substantial reduction in variability among the studies.

**Figure S1:**
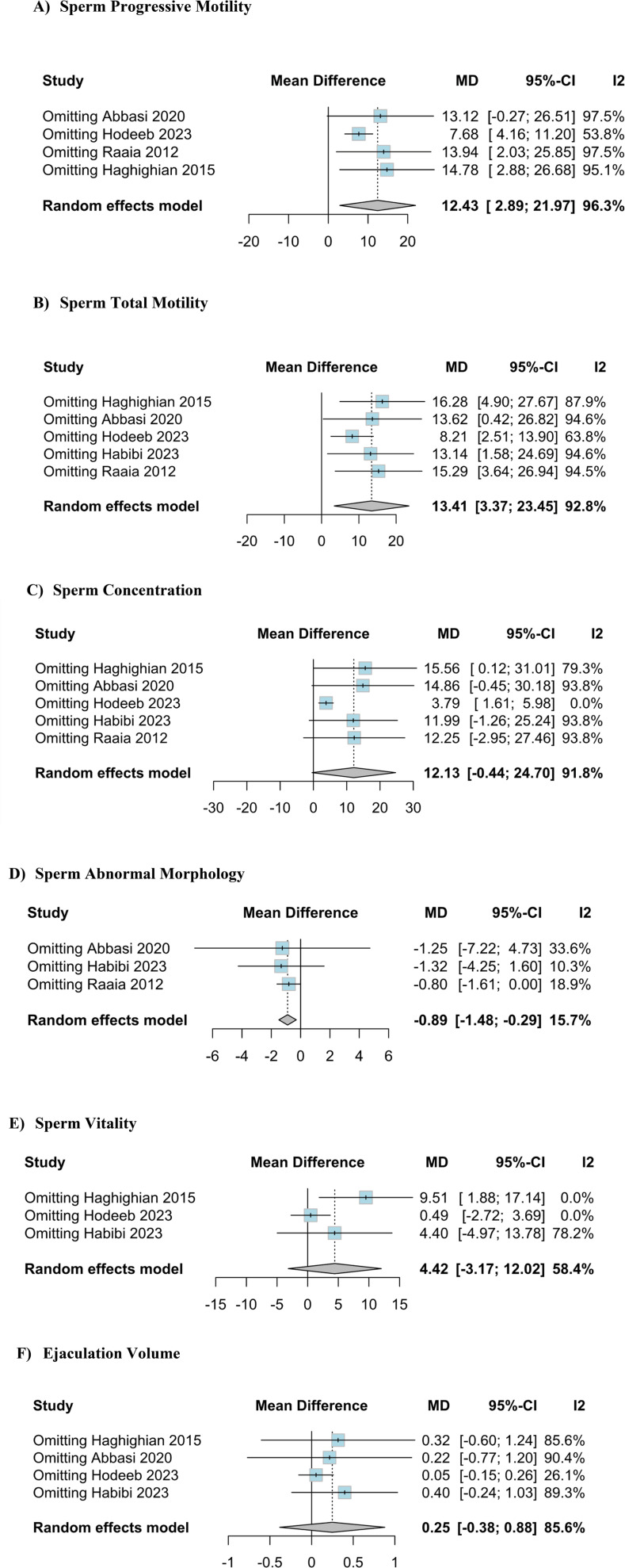
Sensitivity analysis using the leave-one-out method to evaluate variations in sperm parameters.

### Risk of Bias and Certainty of Evidence

Out of five randomized studies, three exhibited a low overall risk of bias (Figure-S2), while two showed moderate risk. The full GRADE assessment is available in supplementary material (Table-S2).

**Figure S2:**
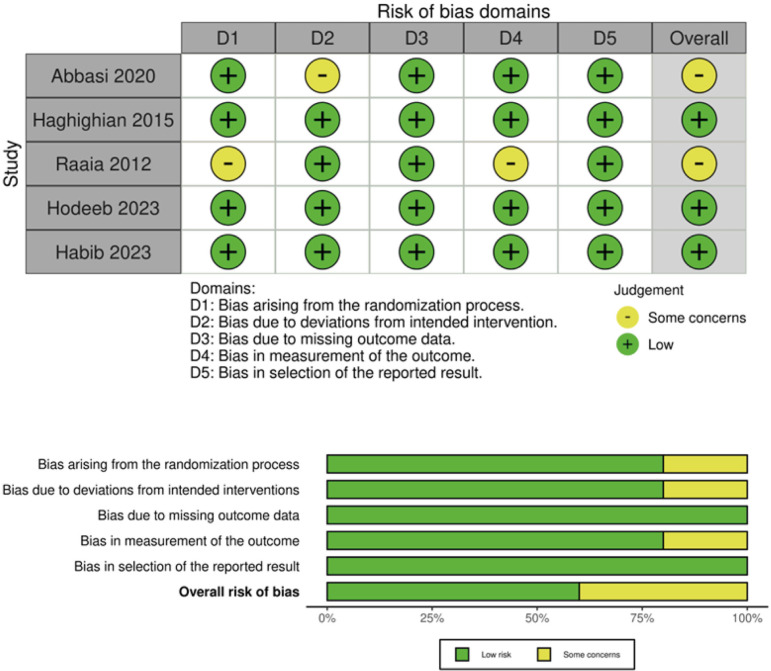
Risk of Bias in Randomized Trials (ROB-2) assessment.

**Table S2:** GRADE assessment of the certainty of evidence across studies. Author(s): Iago Zang Pires, Marília Oberto da Silva Gobbo, Renan Yuji Ura Sudo, Tanize Louize Milbradt, Nilson Marquardt Filho, Gustavo Franco Carvalhal, Carlos Teodosio Da Ros Question: Alpha lipoic acid compared to placebo for increase sperm quality

Certainty assessment							N° of patients		Effect		Certainty	Importance
N° of studies	Study design	Risk of bias	Inconsistency	Indirectness	Imprecision	Other considerations	Alpha lipoic acid	placebo	Relative (95% CI)	Absolute (95% CI)		
**Abnormal morphology**												
3	randomised trials	not serious	not serious	not serious	not serious	none	68	58	-	MD**0.89 lower** (1.48 lower to 0.29 lower)	⨁⨁⨁⨁ !High	CRITICAL
**Ejaculation volume**												
4	randomised trials	not serious	not serious	not serious	not serious	none	107	95	-	MD**0.14 higher** (0.54 lower to 0.83 higher)	⨁⨁⨁⨁ High	IMPORTANT
**Live sperm**												
3	randomised trials	not serious	not serious	not serious	not serious	none	88	73	-	MD**4.42 higher** (3.17 lower to 12.02 higher)	⨁⨁⨁⨁ High	IMPORTANT
**Motility Grade (a + b + c)**												
5	randomised trials	not serious	not serious	not serious	not serious	none	131	119	-	MD**13.49 higher** (3.52 higher to 23.46 higher)	⨁⨁⨁⨁ High	CRITICAL
**Sperm concentration**												
5	randomised trials	not serious	not serious	not serious	not serious	none	131	119	-	MD **11.99 higher** (0.67 lower to 24.66 higher)	⨁⨁⨁⨁ High	IMPORTANT
**Sperm Progressive Motility**												
4	randomised trials	not serious	not serious	not serious	not serious	none	106	107	-	MD**12.43 higher** (2.89 higher to 21.97 higher)	⨁⨁⨁⨁ High	CRITICAL
**Pregnancy rate**												
2	randomised trials	not serious	not serious	not serious	not serious	none	9/49 (18.4%)	3/36 (8.3%)	**RR 2.28** (0.66 to 7.85)	**107 more per 1000** (from 28 fewer to 571 more)	⨁⨁⨁⨁ High	CRITICAL

**CI:** confidence interval; **MD:** mean difference; **RR:** risk ratio

## DISCUSSION

In this systematic review and meta-analysis of five RCTs, comprising 250 patients with a mean age ranging from 28 to 40 years old, ALA was evaluated against sham-therapy. Our analysis demonstrated an increase in both total (p = 0.008) and progressive (p = 0.011) sperm motility, a reduction of sperm abnormal morphology (p = 0.003), and a slight improvement in sperm concentration (p = 0.063). However, no statistical difference was found in pregnancy rates in our pooled analysis, possibly due to the small sample and short follow-up, as well as other outcomes, such as total antioxidant capacity.

Male infertility has a vast range of causes and, as a result, the true incidence of this condition remains unclear (20). There are many options as treatment alternatives (21), including acetyl-L-carnitine, L-carnitine fumarate, glutathione, carnitines, N-acetylcysteine, vitamins E and C, selenium, zinc, and folic acid; among these, antioxidants are the most significant (22).

**Figure 5:**
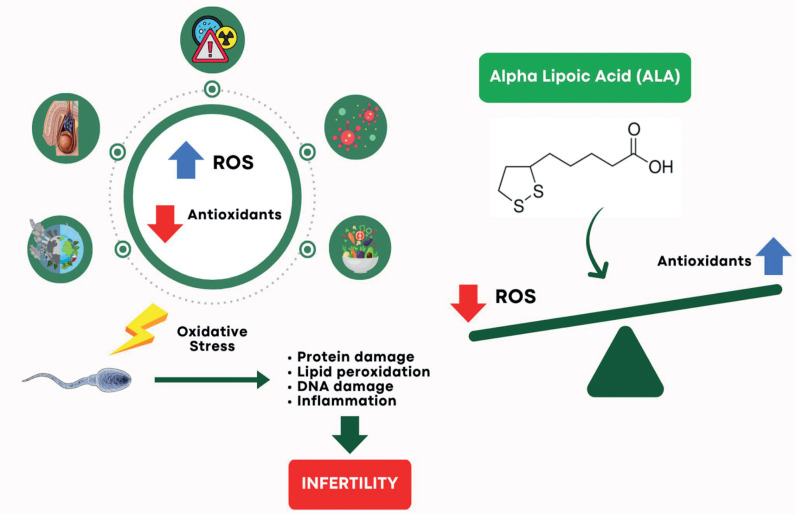
Role of ALA in Balancing Oxidative Stress to Prevent Infertility.

Several studies have investigated the antioxidant effects of ALA on human and animal sperm functions. Previous systematic reviews and meta-analysis evaluated the effects of ALA supplementation on endothelial function (23), metabolic syndrome (24), dyslipidemia improvement (25), glycemic control (26), human infertility (27), central nervous system disorders (28), and obesity (29). Many studies acknowledge the role of Oxidative Stress (OS) in the etiology of male infertility, with nearly 30-80% of infertile men exhibiting high seminal ROS levels (30). Nonetheless, OS mediates the deleterious effects of hypertension, obesity, insulin resistance, mental disorders, and prolonged ejaculatory abstinence periods (31), as well as varicocele (32) and environmental contaminants (33). It can lead to protein damage, lipid peroxidation (34), DNA damage, and inflammation (35), which ultimately reduces sperm quantity and function, contributing to male infertility (11). Figure-5 illustrates the oxidant process.

ALA acts effectively in both lipid and water soluble states, enabling it to penetrate tissues, cells, and organelles, such as mitochondria, thus providing a broad spectrum of action to mitigate the adverse effects of ROS (36). The regeneration of other antioxidants including vitamins E, vitamins C, and glutathione represents another important mechanism of ALA (37). The effects on sperm motility and concentration relies heavily on mitochondrial ATP production, and ALA, assisting in the oxidative decarboxylation metabolism as a mitochondrial coenzyme, enhances membrane capacity by increasing cytochrome C concentrations. This, in turn, boosts ATP availability and ensures a consistent supply of energy for sperm movement (38). The enhancement in oxidative decarboxylation would increase cytochrome C level and therefore improve the mitochondrial membrane potential, function, and biogenesis. Our study could corroborate these findings, ALA improved sperm motility and slightly enhanced the sperm concentration.

There is only one prior systematic review and meta-analysis, which included three studies, comprising 133 patients, and could not assess pregnancy rates, limiting a more precise analysis (39). Our research differs in its larger sample size, which includes 250 patients, providing more reliable data on the improvement of sperm parameters, and the analysis of pregnancy rates.

At the present, ALA is commonly used for patients presenting some degree of peripheral neuropathy (40). As we demonstrated in our study, it also can be used for idiopathic male infertility, the dosage is 600 mg per day, that could be divided two or three times a day. Thus, among the included studies, none reported adverse effects during ALA supplementation.

Our study has limitations. Although the studies analyzed were prospective, blinded, and randomized, only five studies were included, and all trials had small samples, only two studies demonstrated pregnancy rates (17, 18). Also, the longest follow-up obtained was 3 months, making long-term analysis unfeasible. Thus, the results might not be reliable or generalizable to larger populations. Even so, our assessment indicated that the majority were at low risk of bias. The heterogeneity of some results complicates drawing definitive conclusions. To minimize these limitations, we performed a random effects model, a Leave-One-Out sensitivity analysis. The review process itself has limitations, particularly the exclusion of non-English-language articles, which may have introduced language bias.

## CONCLUSIONS

Our meta-analysis comprehending five RCTs suggests strong evidence for ALA supplementation, which was associated with a favorable change in sperm function, especially spermatozoa motility. However, there were no effects on the pregnancy rates, likely due to the small sample size and short follow-up. Nevertheless, ALA should be considered as an adjuvant treatment for patients with idiopathic infertility. Further prospective randomized controlled trials are needed, particularly those including pregnancy rates and longer follow-ups.

## APPENDIX:

### Supplementary Material Search Strategy:

(((((((((((((((((((((spermatozoa) OR (semen)) OR (sperm)) OR (male infertility)) OR (male subfertility)) OR (asthenozoospermia)) OR (asthenospermia)) OR (oligozoospermia)) OR (oligospermia)) OR (oligoasthenozoospermia)) OR (oligoasthenospermia)) OR (oligoasthenoteratozoospermia)) OR (oligoasthenoteratospermia)) OR (OAT)) OR (teratozoospermia)) OR (teratospermia)) OR (spermatogenesis)) AND (oral alpha-lipoic acid)) OR (alpha-lipoid acid)) OR (alpha lipoic acid)) OR (α-lipoid acid)) OR (thioctic acid) AND (randomized trials) OR (randomized control trial) OR (RCT)
